# Selective Susceptibility of Human Skin Antigen Presenting Cells to Productive Dengue Virus Infection

**DOI:** 10.1371/journal.ppat.1004548

**Published:** 2014-12-04

**Authors:** Daniela Cerny, Muzlifah Haniffa, Amanda Shin, Paul Bigliardi, Bien Keem Tan, Bernett Lee, Michael Poidinger, Ern Yu Tan, Florent Ginhoux, Katja Fink

**Affiliations:** 1 Singapore Immunology Network, Agency for Science, Technology and Research, Singapore; 2 School of Biological Sciences, Nanyang Technological University, Singapore; 3 Institute of Cellular Medicine, Newcastle University, Newcastle upon Tyne, United Kingdom; 4 Institute of Molecular Biology, Agency for Science, Technology and Research, Singapore; 5 Division of Rheumatology, University Medicine Cluster, National University Health System, Singapore; 6 Department of Plastic Surgery, Singapore General Hospital, Singapore; 7 Department of General Surgery, Tan Tock Seng Hospital, Singapore; Purdue University, United States of America

## Abstract

Dengue is a growing global concern with 390 million people infected each year. Dengue virus (DENV) is transmitted by mosquitoes, thus host cells in the skin are the first point of contact with the virus. Human skin contains several populations of antigen-presenting cells which could drive the immune response to DENV *in vivo*: epidermal Langerhans cells (LCs), three populations of dermal dendritic cells (DCs), and macrophages. Using samples of normal human skin we detected productive infection of CD14^+^ and CD1c^+^ DCs, LCs and dermal macrophages, which was independent of DC-SIGN expression. LCs produced the highest viral titers and were less sensitive to IFN-β. Nanostring gene expression data showed significant up-regulation of IFN-β, STAT-1 and CCL5 upon viral exposure in susceptible DC populations. In mice infected intra-dermally with DENV we detected parallel populations of infected DCs originating from the dermis and migrating to the skin-draining lymph nodes. Therefore dermal DCs may simultaneously facilitate systemic spread of DENV and initiate the adaptive anti-viral immune response.

## Author Summary

Dengue virus (DENV) is transmitted by mosquitoes with skin as point of entry for the virus. Here, we investigated DENV infection in primary human skin cells and their initial immune response. Using skin from normal human donors for infection with DENV *in vitro* we identified antigen-presenting cells (APCs) as main targets of DENV. Further analysis showed that only distinct subsets of dendritic cells (DCs) and macrophages were infected and efficiently produced viral progeny. Langerhans cells were most susceptible to infection despite lacking DC-SIGN, a previously described DENV receptor. Infection of the other DC subsets and macrophages was also independent of DC-SIGN expression. Genes of the interferon pathway and CCL5, a chemokine attracting immune cells to sites of inflammation, were highly up-regulated in the infected DC subsets. Using a mouse infection model, we showed that murine dermal DCs were also susceptible to DENV and migrated to draining lymph nodes. At the same time infiltrating monocytes differentiated into monocyte-derived cells at the site of infection and became an additional target for DENV *in vivo*. These data demonstrate that DENV differentially infects and activates primary human skin APCs and that infected cell types individually contribute to inflammation and the adaptive response.

## Introduction

*Aedes* mosquitoes are the primary vectors for the transmission of dengue virus (DENV). While probing for blood microvessels from which to feed, the mosquito releases virus-containing saliva into the dermal layer of the skin. Studies using mosquitoes infected with the closely-related West Nile virus showed that more than 99% of the viral particles could be recovered from around the feeding site on mice, indicating that most of the virus is not injected directly into the blood but rather pools in the local tissue [1]. Precisely how such viruses, including West Nile and DENV, then spread to cause systemic infection is currently unknown.

Human skin is composed of an epidermal and a dermal layer, separated by the basement membrane. The epidermis contains keratinocytes and Langerhans Cells (LCs), a specialized type of dendritic cell (DC) that constantly probes for antigen in the most exposed, superficial layer of the skin [2]. Upon detection of pathogens during an infection LCs migrate to draining lymph nodes (LNs) where they contribute to the initiation of T cell responses. Although early studies suggested that LCs were the principal migratory DC initiating T cell responses, more recent findings have demonstrated a key role for LCs in Treg activation and skin homeostasis [3]–[5]. Mice in which LCs have been depleted still generate protective skin-specific T cell responses [6]. The underlying dermis, in contrast, contains fibroblasts as wells as high numbers of immune cells including macrophages, T cells and three subsets of dendritic cells (DCs) [7]–[9]. Additionally, the dermis harbors a dense network of blood and lymphatic vessels [7], through which immune cells and mediators can both enter and exit the tissue. The three dermal DC subsets are distinguished by positive expression of CD1c (a MHC I-related molecule that presents lipids to T cells), CD14 (co-receptor for bacterial lipopolysaccharide) or CD141 (thrombomodulin). CD1c^+^ DCs are the most abundant amongst the three subsets, and following activation in the skin their functional role is to migrate to draining LNs for the initiation of systemic T cell responses [10]. CD14^+^ DCs are less abundant than CD1c^+^ DCs, and were recently shown to be monocyte-derived cells transcriptionally related to macrophages [11] [12]. Skin CD14^+^ DCs have the capacity to activate CD4^+^ T cells and drive their differentiation into T follicular helper cells (Tfh) that support the efficient initiation of antibody responses [10], [13]. CD14^+^ DCs also have the capacity to induce tolerance by promoting the generation of Tregs in the presence of Vitamin D3 [5], [14]. The third and least abundant subset of skin DCs are the CD141^+^ DCs, which also migrate to LNs but specialize in cross-presenting antigen to CD8^+^ T cells [15]. Recently, murine homologs of human tissue DC subsets were identified [9], [15], which raises the possibility of drawing new parallels from findings using murine viral infection models.

In this study we interrogated the host target cells of DENV at the physiological entry site of infection in human skin, to understand their functional relevance in the development of dengue-specific infection and immunity. Our findings demonstrate heterogeneity in susceptibility to dengue virus infection within skin APC subsets in both humans and mice. These results enhance our understanding of the early consequences of dengue virus infection.

## Results

### DENV infects LCs, CD1c^+^ and CD14^+^ subsets of DCs and dermal macrophages from human skin

**Figure 1:**
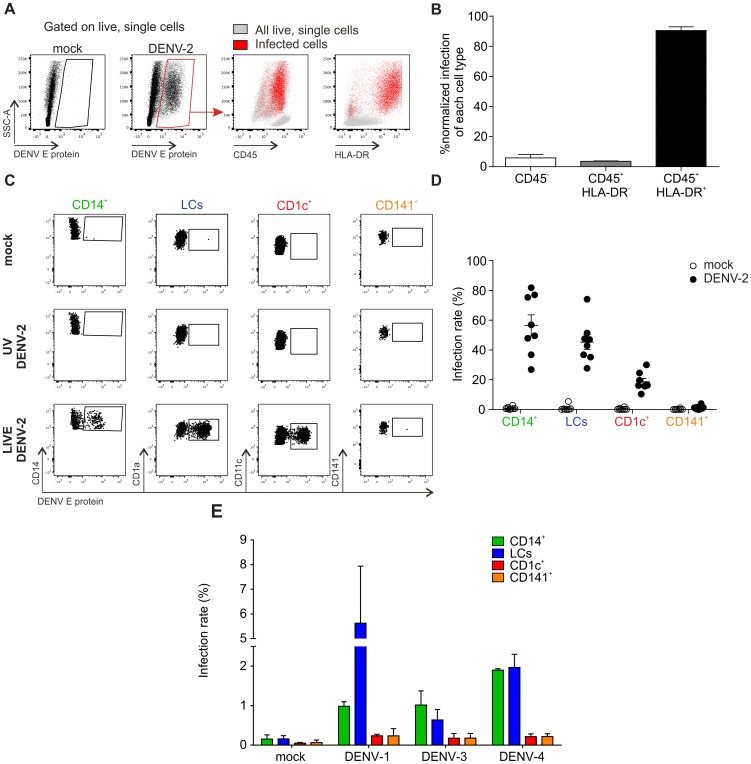
Identification of DENV susceptible cells from healthy human skin samples. (A) Flow cytometry of collagenase-treated healthy human skin incubated with medium (mock) or DENV-2 (multiplicity of infection, MOI 2) for 48 h (left). All cells (grey) were overlaid with infected cells (red) to determine expression of CD45 and HLA-DR. (B) Summary data of infected cells from (A) according to their expression of CD45 and HLA-DR of n = 7 donors, mean ± SEM from 7 independent experiments. (C) Mock, UV-inactivated and LIVE DENV-2 treated HLA-DR^+^ cells from (A) were subjected to surface molecule expression analysis to identify four different subsets of dendritic cells (DCs) (gating strategy shown in Figure S1): CD14^+^ dermal DCs, epidermal Langerhans cells (LCs), CD1c^+^ DCs and CD141^+^ DCs. Infection was quantified by flow cytometry according to detection of DENV E protein. (D) Summary data of (C), each dot represents one donor, mean ± SEM. (E) Cells were exposed to DENV-1, -3 and -4 (MOI 10) for 48 h and presence of DENV E protein detected by flow cytometry, n = 3 donors, mean ± SEM from three independent experiments.

To identify the cell types within human skin that are susceptible to DENV infection we prepared single cell suspensions from healthy skin obtained from mastectomy or abdominoplasty surgery, and exposed the cells to DENV-2 strain D2Y98P [16] at an MOI of 2. After 48 h, flow cytometry was used to characterize the infected cell types by measuring the percentage of cells positive for DENV E protein, which forms part of the viral envelope (Fig. 1). The vast majority (approximately 90%) of cells positive for E protein expressed CD45 and HLA-DR (Fig. 1A and B). This finding excluded significant infection of CD45^−^ keratinocytes, fibroblasts and endothelial cells, which had been reported previously to be possible targets of DENV infection [17], [18]. CD45^+^ HLA-DR^+^ cells include all antigen-presenting cells (APCs) in the skin. To further refine our analysis we employed a previously described gating strategy to distinguish between CD14^+^ DCs, CD1c^+^ DCs, CD141^+^ DCs and LCs ([15] and S1A Figure). Three skin DC subsets were susceptible to DENV-2 infection *ex vivo*: LCs and CD14^+^ DCs were infected most efficiently while CD1c^+^ cells showed a lower infection rate (Fig. 1C). To prove that E protein-positive cells were truly infected and had not only taken up virus particles, cells were also treated with UV-inactivated virus as a control (Fig. 1C). The infection profile for skin DC subsets was reproducible and independent of the skin donor (Fig. 1D). Interestingly, we did not detect infection of CD141^+^ DCs. To test whether there were serotype-specific differences in infection we also exposed single cell suspensions from skin to DENV-1, -3 and -4. These experiments showed that LCs and CD14^+^ DCs were consistently infected at higher rates than CD1c^+^ and CD141^+^ DCs, showing a similar DENV infection profile in human skin cells to DENV-2 (Fig. 1E). Of note, infection with DENV-1, -3 and -4 was less efficient than infection with DENV-2 D2Y98P (Fig. 1A and B), which was expected due to the enhanced viral RNA synthesis capacity of the latter [19]. A less virulent DENV-2 strain (TSV01) showed lower infection rates than D2Y98P, but a similar target cell infection profile (Fig. S1C). In addition to DCs we identified dermal macrophages by flow cytometry based on their auto-fluorescence in the FITC channel [20] (Fig. S1A and B). CD14^+^ DCs and dermal macrophages were infected at similar rates 24 h after infection (Figure S1B), identifying both DCs and macrophages in the skin as potential DENV targets.

As *Aedes* mosquitoes deposit virus-containing saliva in the dermal layer, bypassing the epidermis when probing with their proboscis to search for blood vessels, we explored infection susceptibility upon intradermal delivery of DENV. LCs were not infected when the virus was injected intra-dermally, in contrast to infection of skin single cell suspension (Figure S1C).

### Infected DCs produce high titers of viral progeny and infection promotes cell survival

**Figure 2:**
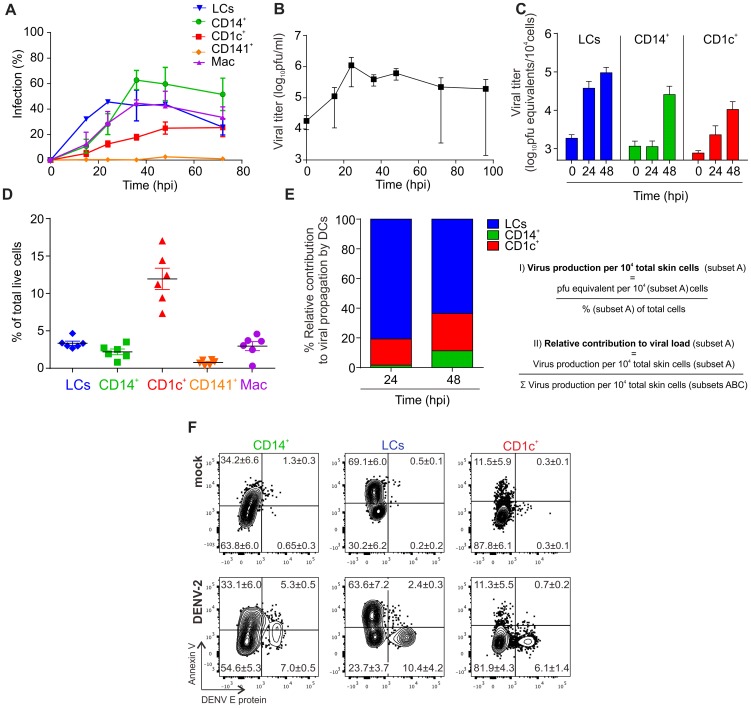
Infection characteristics of DENV in DC subsets from human skin. (A) Cells from collagenase-treated healthy human skin were exposed to DENV-2 at MOI 2 for the indicated times. Presence of DENV E protein in DC subsets was established by flow cytometry. (B) Amount of live virus in supernatants of cells from (A) was quantified by plaque-forming assay and used to calculate titer in plaque-forming units per ml (pfu/ml), n = 3 donors, mean ± SEM from three independent experiments. (C) Sorted DC subsets were infected with DENV-2 at MOI 2 and viral RNA was measured in the supernatant by quantitative real time PCR at 0, 24 and 48 hpi. n = 3, mean ± SEM from three independent experiments. (D) Analysis of skin DC subsets and macrophages by flow cytometry in whole human skin. Each dot represents one donor, mean ± SEM. (E) Results from (C) and (D) were used to calculate the relative contribution of each DC subset to the total viral load at 24 and 48 hpi. (F) Infected DC subsets from skin were stained with Annexin V and labeled for DENV E protein after 24 h of exposure to virus to determine the extent of apoptosis (one representative donor of three), mean % per quadrant ± SEM.

Whereas flow cytometry only detects how much viral protein is expressed in infected cells, production of infectious virus particles by infected cells can be assessed by using a plaque-forming assay or by measuring viral RNA by PCR in the cell culture supernatant. To further characterize the infection kinetics of various skin DC subsets we infected single cell suspensions of human skin with DENV-2 strain D2Y98P at an MOI of 2, analyzed the cells by flow cytometry and cell culture supernatants by plaque assay at 16 h, 24 h, 36 h, 48 h and 72 h after infection (Fig. 2A and B). We found that LCs were infected most rapidly but that their infection frequency plateaued after 24 h. The infection kinetics were delayed for CD14^+^ DC and CD1c^+^ DCs but peaked to similar infection levels as LCs after 36 hrs. CD141^+^ DCs were resistant to infection throughout the entire time course (Fig. 2A). Macrophage and CD14^+^ DCs showed similar infection kinetics but overall infection levels were lower for macrophages compared to CD14^+^ DCs after 36 hrs. Virus titers measured in the supernatant of infected total skin cells increased to a peak at 24 h after infection before declining. Only one out of three donors showed extended virus production until 96 h after infection (Fig. 2B). To test whether and how much virus was produced by individual DC subsets, skin cells were sorted, infected, and viral RNA was extracted from the cell culture supernatants for qRT-PCR analysis. Macrophages were not viable after sorting and could not be included in this experiment. 24 h after infection, we observed the highest titers in LCs compared to the other two subsets, which showed only little (CD1c^+^) or no increase in secreted virus (CD14^+^) at this time point (Fig. 2C). At 48 h, all infected DC subsets showed an increase in virus production, whereby LCs remained the most efficient producers. To determine the relative viral load contributed by each DC subset we first determined the relative numbers of each subset in digested whole skin (Fig. 2D) and then calculated their relative contribution (Fig. 2E). This analysis revealed that LCs were the main contributors of viral load produced by DCs, followed by CD1c^+^ DCs, which were present in higher numbers than CD14^+^ DCs.

We next assessed if DENV infection resulted in cell apoptosis. Using AnnexinV staining we found that a significantly lower proportion of infected CD1c^+^ DCs and LCs were apoptotic compared to their non-infected counterparts in the same tissue culture well suggesting that infection per se did not induce APC apoptosis at either 24 h after infection (Fig. 2F) or up to 90 h after infection (Table S1). CD14^+^ DCs appeared to be more susceptible to apoptosis following DENV infection (Fig. 2F). Prolonged survival of infected cells might represent an effective strategy of the virus to maximize the time for production of viral progeny.

In summary, we found that infected human skin DCs were capable of producing significant amounts of DENV in the absence of increased levels of apoptosis in infected cells.

### Infection of skin APC subsets is independent of DC-SIGN

**Figure 3:**
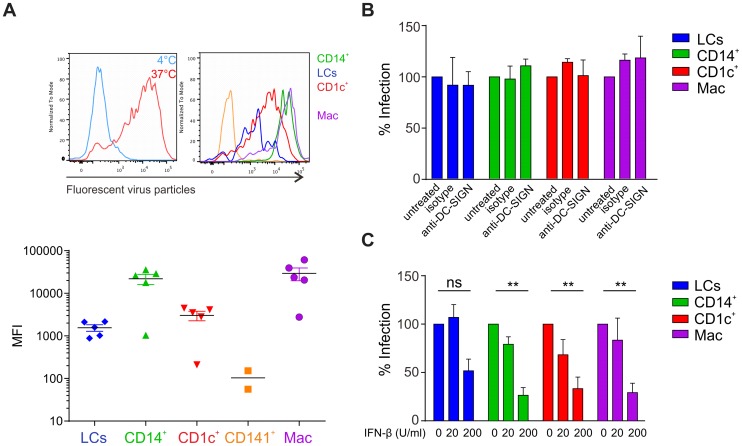
Infection of primary skin DCs is independent of DC-SIGN. (A) DEPC-inactivated, fluorescently-labeled DENV-3 was added to skin cells for 2 h at 4°C or 37°C (left) before flow cytometric analysis of viral uptake. Representative results of one donor are shown (right) and summary data are depicted as mean fluorescence intensity (MFI) at 37°C minus MFI at 4°C (bottom), each dot represents one donor, mean ± SEM. (B) APC subsets were incubated with DC-SIGN blocking antibody for 1 h before infection for 24. n = 3 donors, Mean ± SEM from two independent experiments. (C) Total skin cells were treated with IFN-β for 24 h before infection for 24 h. Infected APC subsets were analyzed by flow cytometry. n = 4, mean ± SEM from three independent experiments.

We next assessed whether the differential infection rates of skin APCs could be explained by variations in individual cell types' ability to take up the virus. After confirming that inactivated, fluorescently labeled virus was still able to bind to host cells (Figure S2A), virus uptake was measured at 37 degrees, whereas lack of uptake at 4 degrees served as negative control (Fig. 3A). 2 h after adding inactivated, fluorescently-labeled virus the highest virus uptake rate was observed in CD14^+^ DCs and dermal macrophages, which was in line with efficient infection. LCs, however, showed a relatively lower viral uptake activity but were still efficiently infected. This was even more surprising with regards to the expression pattern of well-described host receptors for viral binding and infection DC-SIGN (CD209) and mannose receptor (CD206) [21] [22], [23], which were absent on LCs [24] (Figure S2B). The phosphatidylserine receptor Axl was recently described as alternative virus-binding receptor [25]–[27] and is expressed on LCs [28]. However, we detected negligible levels of Axl on the surface of skin DC subsets isolated by collagenase-digestion or spontaneous migration from skin explants *ex vivo*. DC-SIGN was expressed on CD14^+^ DCs but not on CD1c^+^ or CD141^+^ DCs, whereas CD206 was expressed on CD14^+^ and CD1c^+^ DCs (Figure S2B).

At least for dermal DCs, expression of these two receptors could therefore explain the more efficient infection of CD14^+^ DCs compared to CD1c^+^ DCs and the absence of infection in CD141^+^ DCs. To further study the relevance of DC-SIGN for infectivity, we first confirmed that DENV-2 D2Y98P did bind to DC-SIGN by incubating DC-SIGN-expressing U937 cells in the presence or absence of DC-SIGN blocking antibody (Figure S2C). Blocking of DC-SIGN on skin APC subsets had no effect on infection rates, suggesting that other receptors utilized by DENV might be more relevant than DC-SIGN on primary skin DCs (Fig. 3B).

Since infection rates by the individual APC subsets might be affected by differential sensitivity to IFN, we pre-treated total skin cells with IFN-β and detected infection rates of APC subsets at 24 h by flow cytometry. Increasing concentrations of IFN-β had a significant inhibitory effect on infection in CD14^+^ DCs, CD1c^+^ DCs and macrophages but not in LCs (Fig. 3C). This suggests that LCs are less sensitive to IFN, allowing the virus to replicate efficiently.

Overall, infection of skin DC subsets did not strictly correlate with the expression of DC-SIGN, mannose receptor or Axl, while the extent of virus particle uptake only correlated with infection in dermal APCs, and not in LCs. These findings suggested that additional cell-inherent parameters including IFN-β susceptibility determined the observed DENV tropism for distinct skin APC subsets.

### DENV infection negatively regulates skin DC allostimulatory potential but efficiently activates type I IFN responses

**Figure 4:**
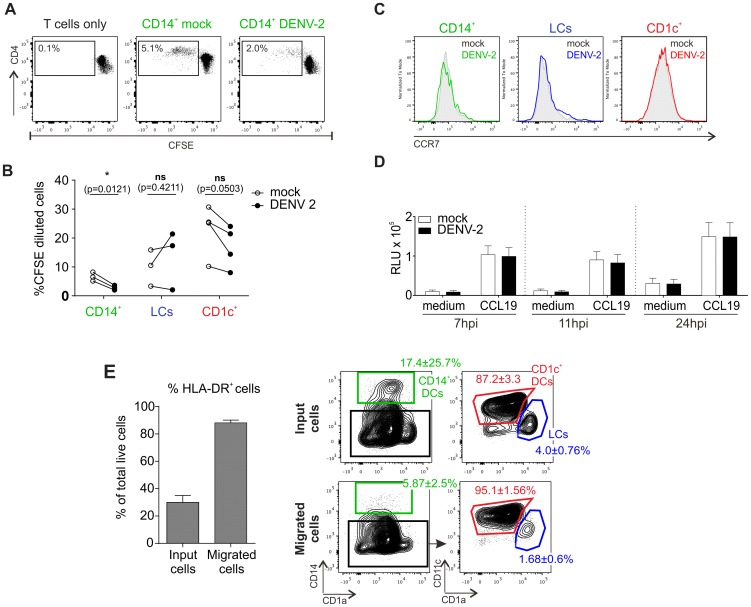
T cell stimulation capacity of DENV-exposed DC subsets and their migratory response towards the chemokine CCL19. (A) Co-culture of sorted infected DCs with allogeneic, CFSE labeled CD3^+^ T cells (ratio 1∶10). Proliferation was measured after 5 days by flow cytometry, one representative result for CD14^+^ dermal DCs is shown. (B) Summary data from all three susceptible DC subsets are shown, each line represents one donor. Statistical analysis was performed using paired two-sided t-test, *p<0.05; ns, not significant, p-values are indicated for each DC subset (C) CCR7 expression on non-treated and DENV-2-exposed DCs at 24 hpi. One representative experiment of three is shown. (D) Skin cell migration was assessed using a 5 µm pore-sized membrane (see Methods Section) with either medium alone or CCL19 (20 ng/ml) in the bottom chamber. Cells were allowed to migrate for 2 h at 37°C before CellTiter Glo activity was measured (RLU, relative light units). Composite data of 4–6 donors is shown, mean ± SEM from 4 independent experiments. (E) Whole skin cells were analyzed by flow cytometry before and after migration towards CCL19. Migrated cells (in the lower well of the chemotaxis plate) were enriched in HLA-DR^+^ cells compared to input cells (left graph). Migrated HLA-DR^+^ cells were enriched in CD1c^+^ DCs and LCs, but not CD14^+^ DCs (right graph; non-infected cells are illustrated; similar results were obtained for infected cells). n = 3 donors, mean ± SEM from three individual experiments.

To evaluate if DENV infection affected T cell stimulatory function of skin DCs, we tested the capacity of the different skin DC subsets infected with dengue virus to stimulate proliferation of allogeneic T cells (Fig. 4A and B). Sorted infected DC subsets were incubated with allogeneic CD3-sorted CFSE-labeled T cells for 5 days before measurement of CD4^+^ and CD8^+^ T cell proliferation by flow cytometry (Fig. 3A and Table S2). DENV-infected CD14^+^ DCs were less efficient at inducing CD4^+^ T cell proliferation compared to their non-infected counterparts (Fig. 4A). This is in keeping with previous observations of poor T cell proliferative responses when PBMCs from dengue infected patients were stimulated with PHA [29]. The defect could be restored by the addition of IL-2 or gamma-irradiated PBMCs from healthy donors, suggesting that APCs but not T cells were impaired in patients [29]. Moreover, DENV infection of monocyte-derived DC (moDCs) inhibited their maturation and their capacity to induce proliferation in allogeneic bulk T cells [30], [31], but not sorted naïve CD4^+^ T cells [32], [33]. In contrast to CD14^+^ DCs, infection of CD1c^+^ DCs and LCs did not impair their capacity to induce allogeneic CD4^+^ T cell proliferation, showing that DENV-mediated inhibition of T cell proliferation was DC subset specific and not a direct generic effect of the virus either on DCs or T cells (Fig. 3B). However, infection of DC subsets had no effect on their capacity to induce CD8^+^ T cell proliferation (Table S2).

We next tested whether infection of skin DCs was likely to have an impact on their capacity to migrate towards the chemokine CCL19, which is expressed in the T cell zones of LN follicles to attract CCR7-expessing migratory cells [34]. CCR7 expression levels were comparable between DC subsets exposed to DENV for 24 h and those treated with medium alone (Fig. 4C), and a functional chemotaxis assay performed at different time points confirmed that DENV infection did not have an impact on the migration of skin DCs *in vitro* as infected cells migrated equally compared to non-infected DCs (Fig. 4D). CD1c^+^ and LCs migrated more efficiently than CD14^+^ DCs in both conditions (Fig. 4E).

**Figure 5:**
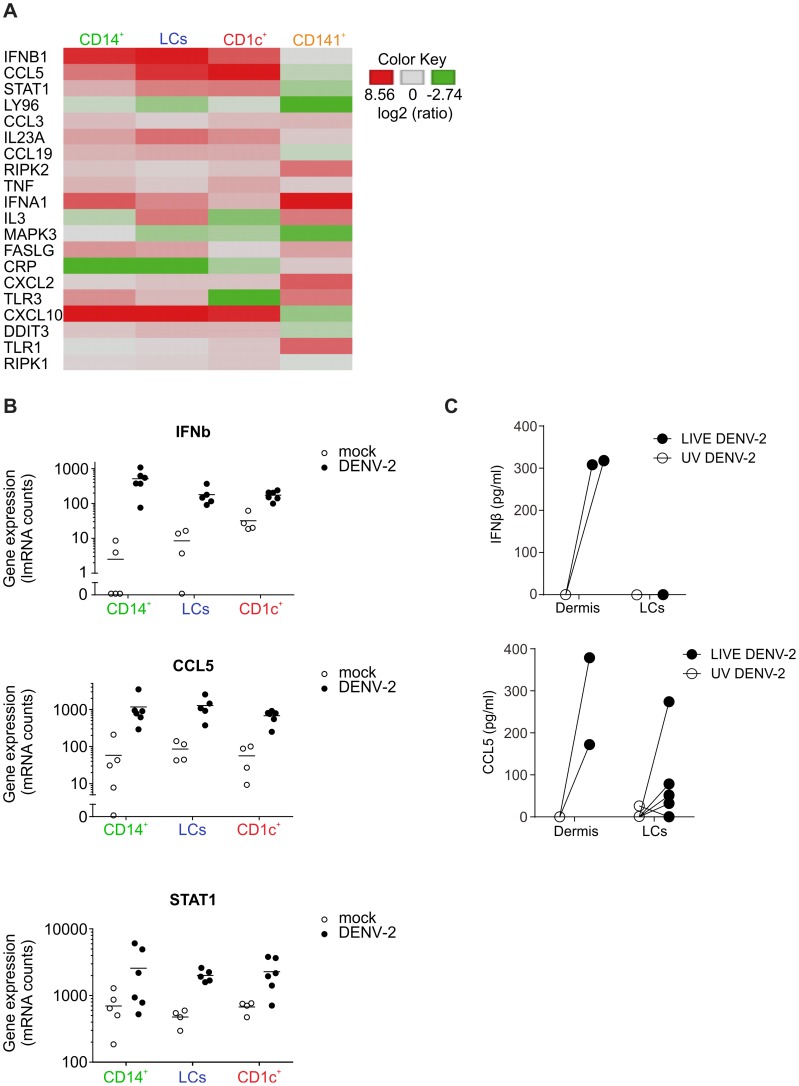
Heatmap of differentially regulated genes in DENV infected skin DC subsets. (A) Nanostring gene expression analysis of sorted human skin DC subsets exposed to DENV-2 (MOI 2) for 24 h. (B) Summary data of three genes that were significantly elevated by DENV exposure in CD14^+^, CD1c^+^ DCs and LCs cultures. Each dot represents one donor. (C) Protein levels of IFN-β and CCL5 produced by dermal cells and by LCs stimulated with UV-inactivated and LIVE DENV-2 for 48 h and measured by ELISA, each dot represents one donor.

To assess the effects of DENV infection on skin DC function, we detected the effects of DENV exposure on the transcription of 184 inflammatory and immune response genes in skin DC subsets. Sorted skin DCs were infected with DENV-2 D2Y98P for 24 h and the mRNA transcripts present in cell lysates were quantified by Nanostring (Fig. 5A). In these experiments, the mean infection rates were 28.1% for CD14^+^ DCs, 39.5% for LCs and 12.5% for CD1c^+^ DCs. Transcription of IFN-β, STAT-1 and CCL5 was significantly up-regulated in all APC subsets upon dengue virus infection. The greatest changes in expression occurred in the CD14^+^ DCs (Fig. 5A). Of note, CD141^+^ DCs did not up-regulate early antiviral genes compared to the other subsets, suggesting that IFN response induction was not responsible for resistance to DENV infection. Up-regulation of IFNA1 gene expression in CD141^+^ cells was not statistically significant and only observed in two out of four donors studied (Fig. 5A). Expression of IFN-β and CCL5 48 h after infection was confirmed by ELISA and was not seen in UV-DENV treated cells, showing that viral replication was necessary to induce innate immune gene up-regulation (Fig. 5B). However, these experiments could not distinguish between gene expression in infected and non-infected cells, which might both contribute to the total gene up-regulation.

Taken together, our data demonstrated that DENV virus infection of human skin DC subsets differentially affected their allogeneic T cell stimulatory capacity. Moreover, primary human skin DCs had the capacity to initiate IFN transcription upon viral infection. In addition, the rapid induction of inflammatory genes such as CCL5 could attract innate immune cells to clear local infection and possibly increase migration of DCs to draining LNs for the initiation of the adaptive response.

### Infected DC subsets in mouse skin are the counterparts of infected DCs in human skin

**Figure 6:**
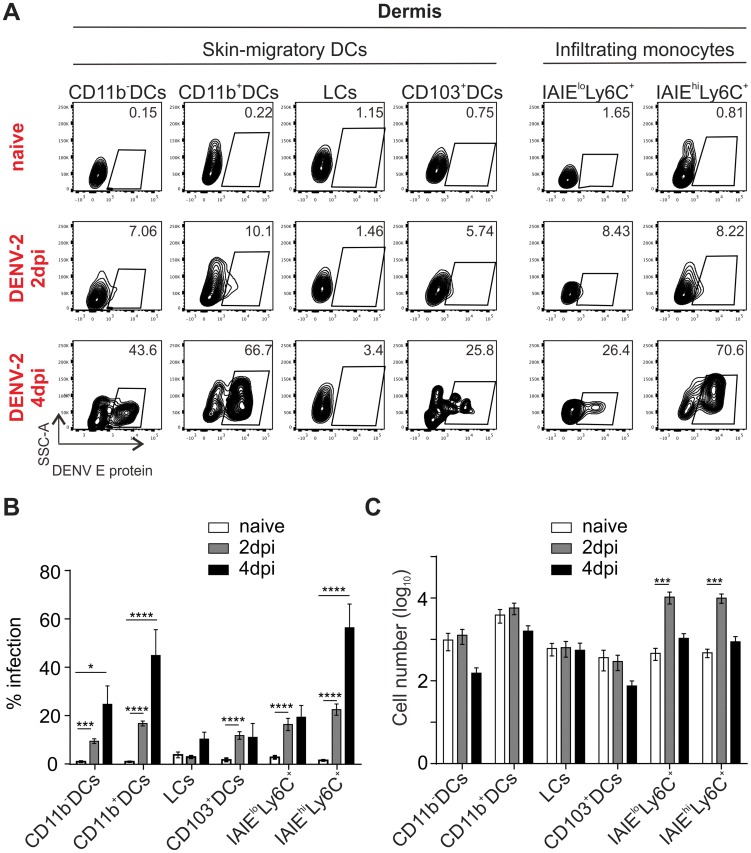
*In vivo* infection of migratory DCs in the skin of IFNAR−/− mice. (A) Mice were inoculated intra-dermally (i.d) with 1×10^6^ pfu of DENV-2 D2Y98P/ear, and ears were harvested 2 and 4 days post infection. DENV E protein expression was detected by flow cytometry of skin-resident DC subsets and infiltrating monocytes (Gating strategy to identify DC subsets/monocytes shown in Figure S2) (B) Summary data of (A), 4 to 5 mice (taking the average of two ears/mouse) per group pooled from two independent experiments, mean ± SEM, analyzed with one-way ANOVA followed by Tukey's multiple comparison test, *p<0.05; **p<0.01; ***p<0.001; ****p<0.0001; non-significant differences are not indicated (C) Quantification of DCs isolated from ears at 2 and 4 dpi to address infiltration and migration of different cell subsets. Same conditions as in (B).

Having identified the cell types in human skin that are able to be infected by DENV, we next wanted to understand the *in vivo* consequences of dengue infection on ensuing functional responses. We recently identified the functional murine homologs of human tissue DC subsets [15], which enabled us to exploit a murine model of dengue infection to interrogate skin APC susceptibility to DENV infection, their LN migratory capacity and the contribution of recruited inflammatory myeloid cells upon DENV infection. As wild-type mice are not susceptible to DENV infection [35], we used interferon-α/β-receptor knock-out (IFNAR) mice, which show disease symptoms and clinical parameters comparable to dengue patients following DENV infection [36]. Mice were infected intra-dermally with 10^6^ pfu of DENV-2 in a volume of 10 ul into each ear. After two and four days mice were sacrificed and we prepared single-cell suspensions from their ears for flow cytometry analysis of infected cell populations staining positively for DENV E protein. The gating strategy (Figure S3A) allowed us to differentiate between dermal CD11b^+^ DCs (homolog of human CD1c^+^ DCs), CD11b^−^ DCs, CD103^+^ DCs (homolog of human CD141^+^ DCs), LCs, MHC class II (IAIE)^hi^ Ly6C^+^ monocyte-derived cells and MHC class II (IAIE)^−^ Ly6C^+^ inflammatory monocytes [37], [38]. CD11b^−^ and CD11b^+^ dermal DCs were frequently infected, reaching infection levels of approximately 20% and 50% respectively by day 4 post-infection (Fig. 6A and B). In contrast, by day 4, LCs were not highly infected and similarly CD103^+^ DCs showed a low level of infection in the region of 10% of cells by day 2 (Fig. 6B). In addition to skin-resident DCs, we found high infection rates in infiltrating Ly6C^+^ cells in the skin two days after infection (Fig. 6A), with a marked increase in infection particularly in the Ly6C^+^IAIE^+^ population on day four after infection (Fig. 6B). In contrast to *ex vivo* human skin cells, we also identified a substantial population of infected CD45^−^ cells in the dermis, but not in the epidermis (Figure S3B). Quantification of total cell numbers 2 and 4 days after infection showed a decrease, although not significant, of CD11b^−^ DCs, CD11b^+^ DCs and CD103^+^ DCs, two days after infection. This was likely due to cell death rather than LN migration as the increase in numbers of migrated cells in draining LNs was already observed at day 2 after infection (see following paragraph). In contrast to dermal DCs, LC numbers remained constant (Fig. 6C).

The murine *in vivo* model also permitted analysis of cells recruited into skin upon intradermal inoculation of DENV. We observed a more than ten-fold increase in the number of inflammatory monocytes (Ly6C^+^IAIE^−^) and monocyte-derived cells (Ly6C^+^IAIE^+^) in the ears of mice within two days of infection. This rise was followed by a rapid decline four days after infection, which may be due to cell death or due to down-regulation of Ly6C expression on activated monocyte-derived cells. Ly6C^−^ monocyte-derived cells could not be distinguished from CD11b^+^ DCs and it was difficult to assess whether there was a relatively smaller decline in this population due to possible parallel effects of cell death and new formation of monocyte-derived cells (Fig. 6C).

Taken together, functionally-equivalent dermal DC subsets appeared to be infected in both humans and mice, with the exception of CD103^+^ cells, which were infected in mouse skin, although at much reduced numbers compared to other subsets, whereas their counterpart in human skin were not infected in our *ex vivo* experiments. In addition, *in vivo* experiments revealed a massive infiltration of Ly6C^+^ monocyte-derived cells, identifying these cells as potentially important infection targets during natural infection.

### Infected skin DCs efficiently migrate to the draining lymph node

**Figure 7:**
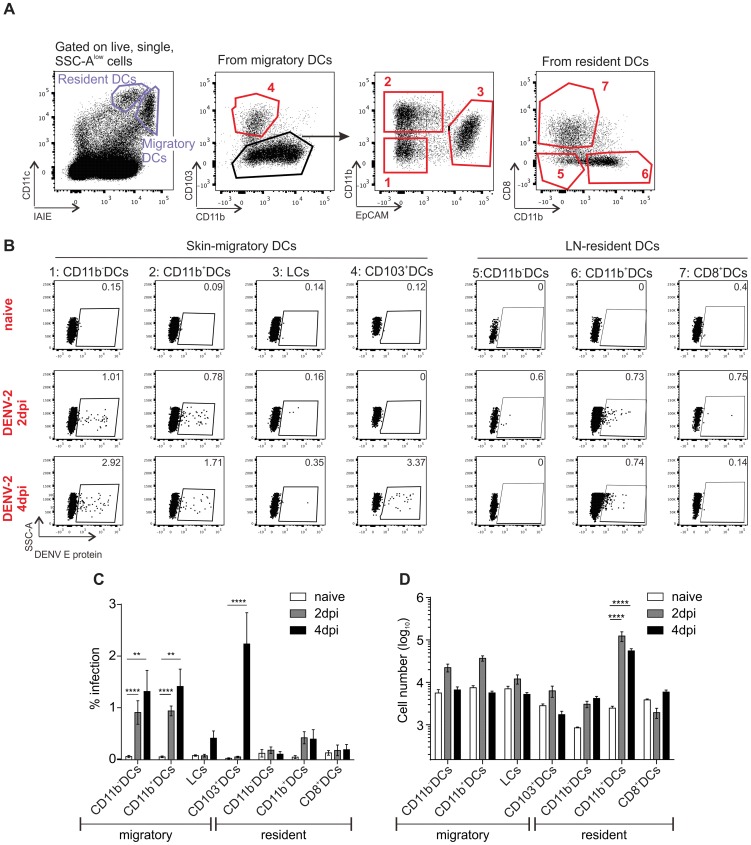
*In vivo* infection of resident and migratory DCs in the skin-draining lymph node of IFNAR^−/−^ mice and their migratory behavior. (A) Gating strategy to identify lymph node (LN)-resident and migratory DCs in the skin-draining LN of IFNAR^−/−^ mice. Resident DC subsets are either CD8^+^ (1), CD11b^−^ (2) or CD11b^+^ (3), while migratory DC subsets incorporate those found in the skin: CD103^+^ (4), CD11b^−^ (5), CD11b^+^ (6) and Langerhans Cells (LCs, 7). (B) Mice were inoculated i.d. with 1×10^6^ pfu of DENV-2 D2Y98P/ear and skin-draining lymph nodes (LN) were harvested 2 and 4 days post infection (dpi). Presence of DENV E protein was established by flow cytometry in both LN-resident and migratory DC subsets. (C) Summary of data from (B), 4 to 5 mice (average of two LN/mouse) per group pooled from two independent experiments, mean ± SEM, analyzed with one-way ANOVA followed by Tukey's multiple comparison test, *p<0.05; **p<0.01; ***p<0.001; ****p<0.0001; non-significant differences are not indicated. (D) Quantification of DCs isolated from LNs at 2 and 4 dpi to address infiltration and migration of different cell subsets. Same conditions as in (C).

It was important to know which cells had the capacity to migrate to draining LNs for the initiation of adaptive immune responses and the ensuing immune memory, and whether those cells carried infectious DENV with them. Cell suspensions were made from ear-draining LNs of infected mice at days 2 and 4, and analyzed by flow cytometry for DENV E protein, with immigrant and resident DC discriminated based on CD11c and MHC Class II (IAIE) expression [39], [40] (Fig. 7A–C). Amongst DCs migrated from the skin, CD11b^−^ and CD11b^+^ DCs, but not LCs were infected. Despite the low CD103^+^ DC infection rate compared to CD11b^+^ DC in the skin, more infected CD103^+^ DCs than CD11b^+^ DCs were observed in the draining LN at day 4 (Fig. 7C). The relative abundance of CD103^+^ DCs in the draining LN compared to other infected subsets migrating from skin, suggests a significant role for this subset in T cell activation at later time points of infection. The LN-resident counterparts of CD103^+^ cells, CD8^+^ DCs, were not infected at the time points tested, suggesting that virus is transported from the skin via DCs and that little virus reaches the LN directly via the lymphatics to infect LN-resident DCs or these cells were not susceptible to infection at this stage (Fig. 7B and C). Similarly, LN-resident CD11b^+^ DCs were also not infected. However, the absolute number of (non-infected) LN-resident CD11b^+^ DCs was significantly greater in infected compared to non-infected mice (Fig. 7D).

In summary, infection of LN-resident DCs was negligible in contrast to active infection of skin APCs within the first 4 days after intradermal DENV delivery. This finding suggested that dermal DCs migrating from the skin to draining LNs were efficient carriers of infectious DENV and implicates them as likely initiators of systemic immunity. Our results further indicated that CD11b^+^ dermal DCs (the equivalents of human CD1c^+^ DCs) might be important to trigger early adaptive anti-DENV T cell responses.

## Discussion

**Figure 8:**
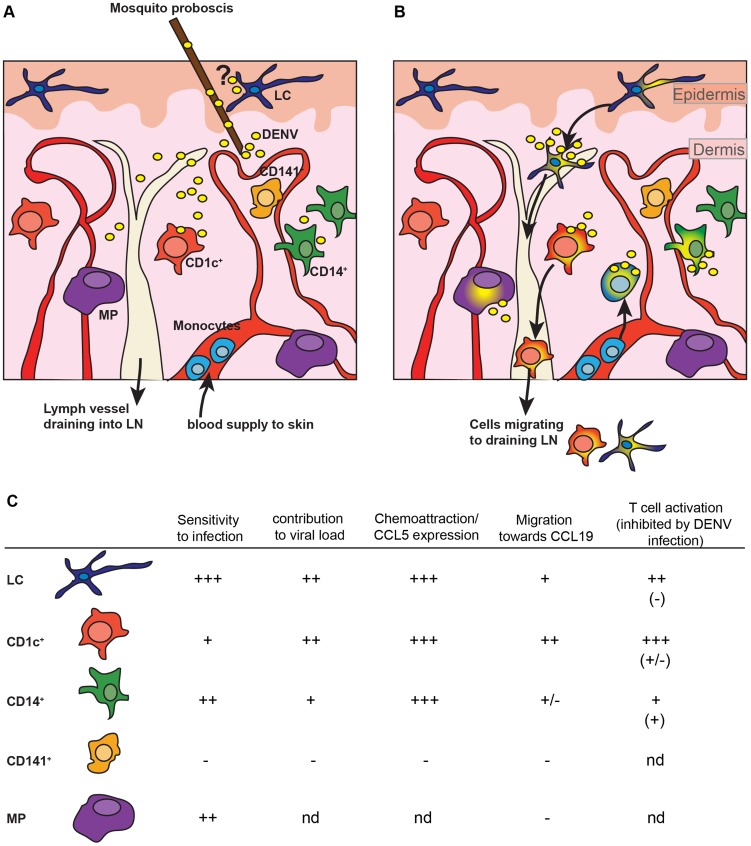
Model for skin dengue infection. (A) The mosquito searches for blood vessels in the dermis and thereby releases saliva that contains virus. The proboscis bypasses the epidermis. The question mark indicates that it is not clear whether during the process of probing virus is also released into the epidermis. (B) Virus in the dermis infects CD1c^+^ and CD14^+^ dermal DCs and macrophages (MP), but not CD141^+^ DCs. Infected CD1c^+^ DCs and possibly LCs migrate to draining LNs to initiate the adaptive immune response. Based on their non-migratory behavior *ex vivo* (Fig. 3E and [12]) CD14^+^ DCs are not expected to migrate to draining LNs. Based on mouse studies, monocyte-derived cells that infiltrate into the skin are infected efficiently (Fig. 6) and contribute to the local and systemic immune response. (C) Table summarizing the role of skin APCs during infection with DENV using single cell suspensions.

The aim of this study was to characterize the cellular targets of DENV infection in human skin, and the consequences of APC infection on systemic infection and the induction of a protective immune response (see model, Fig. 8). Previous studies have focused on the role of LCs in DENV infection, but dermal DCs were not evaluated [41]. DENV is most likely injected into the dermis by its mosquito vector [42] and we provide evidence that human dermal APCs can also be efficiently infected by DENV. In fact, when DENV is injected intradermally *ex vivo* or in mice, LCs are not infected efficiently and the functional relevance of natural LC infection therefore remains unclear. Virus might come into contact with LCs during the process of probing even though the mosquito's proboscis bypasses the epidermis and LCs located there. Alternatively, LCs that migrate through the dermis towards draining LNs might be infected en route, although the number of spontaneously migrating LCs in healthy skin is very small [43]. The suspension cell infection model cannot solve this question and further experiments, ideally with infected mosquitoes injecting the virus into the skin of mice or other animal models, will be required to validate our findings.

Interestingly, we found that DC-SIGN expression did not correlate with infection and blocking of the receptor did not reduce the rate of infection in cells expressing DC-SIGN. Monocyte-derived DCs (moDCs) [44], which represent an easily accessible and hence useful model to study DCs *in vitro*, express high levels of DC-SIGN. The majority of primary DC subsets found in blood, skin and lymph nodes, however, does not express DC-SIGN when analyzed *ex vivo* [45], [46]. It was previously shown that DC-SIGN facilitates attachment to the cell rather than mediating viral endocytosis *per se* and that a potentially unknown *bona fide* receptor is required for viral entry [47]. We therefore speculate that DC-SIGN is one of several receptors expressed on primary DCs that facilitate attachment and viral entry [48].

In mice, CD11b^+^ DCs, which are the functional homologs of human CD1c^+^ DCs, had the highest infection rate. In addition, recruited inflammatory Ly6C^+^ monocyte-derived cells into skin were also efficiently infected (Figure S3, population 6). Based on the findings in mice, we speculate that human blood monocytes infiltrate the skin upon infection, similar to Ly6C^+^ mouse monocytes, and represent an additional target population for the virus. Addressing this question in humans *in vivo* is a challenge, as skin biopsies from patients from the site of the mosquito bite would have to be analyzed.

Human steady state CD14^+^ dermal DCs were efficiently infected. This subset expressed low levels of CCR7 (Fig. 4C) and is unlikely to migrate efficiently to draining LNs (Fig. 4E and [12]). However, CD14^+^ cells are efficient activators of memory T cells [12] suggesting a role in local tissue responses, particularly during secondary infection when DENV-specific T cells may be present in the skin. Alternatively, infection of CD14^+^ DCs could affect their capacity to induce regulatory CD4 T cells in the skin, a function that has been associated with CD14^+^ DCs and not with CD1c^+^ DCs [5], [14]. We were unable to establish in the *in vivo* model if infiltrating Ly6C^+^IAIE^+^ cells migrated from the skin to the draining LNs as Ly6C could be downregulated during LN migration as shown for a West Nile virus murine infection model [49].

Despite the obvious similarities between human and mouse skin infection targets there were also notable differences: firstly, we observed a substantial population of CD45^−^ infected cells in mouse, but not human, skin. This is challenging to interpret, but the lack of the IFNα/β receptor in these mice could have affected the susceptibility of non-hematopoietic cells. We showed recently that the absence of the IFNα/β receptor on CD11c^+^ and LysM^+^ expressing cells alone was sufficient to replicate the DENV-susceptibility phenotype of IFNAR^−/−^ mice with regards to viremia and survival [35], though this does not directly exclude the possibility of some alterations to viral tropism within the model. The second difference between human and mouse was that human CD141^+^ DCs remained uninfected up to 72 h after infection *ex vivo*, whereas CD103^+^ DCs in mice were infected at day four after infection. It could be that CD141^+^ DCs may become susceptible at later time points after infection *ex vivo* or that CD141^+^ DCs are infected in the context of a natural infection. It remains to be addressed whether the reason for this discrepancy relates to species-specific differences in the antiviral response. For human CD141^+^ DCs, infection-induced up-regulation of IFN-β and STAT-1 gene expression was low compared to CD14^+^ DCs and CD1c^+^ DCs (Fig. 4), which might also reflect the lack of infection of the cells at these time points. The induction of transcription of the monocyte-attracting cytokine CCL5 in human cells fits nicely with our observation of infiltrating monocyte and monocyte-derived cells in mice, making it likely that monocyte-derived cells are similarly attracted to the site of infection in humans [50]. IFN signatures and CCL5 were previously found to be up-regulated in microarrays of dengue patients' PBMCs [51], [52] and in the serum [50], whereby higher expression seemed to be associated with less severe disease.

We demonstrate here that human skin DCs are likely to be important targets for DENV infection *in vivo*. The observations in mice suggest that skin dermal DCs were also likely to transport infectious virus to draining LNs, providing a shuttle for the virus to potentially establish further sites of infection, and to efficiently activate a systemic immune response. Our data suggest that intra-dermal or gene-gun inoculation of live-attenuated dengue vaccine candidates would likely target the most physiologically relevant DC populations within the dermis and thereby potentially stimulate the efficient establishment of a systemic immune response.

## Materials and Methods

### Ethics statement

Healthy human skin tissue was obtained from mastectomies or abdominoplastic surgery. The studies were approved by the respective institutional review boards (National Health Group Domain Specific Review Board (NHG DSRB 2012/00928) and Singhealth Centralized Institutional Review Board (CIRB 2011/327/E), respectively) and patients gave written informed consent. All skin samples were processed on the day of surgery. Blood from anonymous healthy human donors was received from the blood bank at the National University Hospital of Singapore and blood donors gave written informed consent. The study was exempted from full IRB review by the Institutional Review Board of the National University of Singapore (NUS-IRB) since anonymous samples were used.

Mouse experiments were conducted according to the rules and guidelines of the Agri-Food and Veterinary Authority (AVA) and the National Advisory Committee for Laboratory Animal Research (NACLAR), Singapore. The experiments were reviewed and approved by the Institutional Review Board of the Biological Resource Center, Singapore (IACUC protocols 100566 and 120801).

### Infection of mice

IFN-α/β receptor-deficient mice (IFNAR^−/−^), on a C57BL/6 background, were infected with 2×10^6^ pfu of D2Y98P via the intradermal (i.d.) route in the ears using a 33-gauge needle and a microsyringe (Nanofil). Naïve mice served as control.

### Cell isolation and culture

For isolation of human skin cells 300 µm dermatome sections were incubated in RPMI+10%FCS (BioWest) containing 0.8 mg/ml collagenase (Type IV, Worthington-Biochemical) and 0.05 mg/ml DNase I (Roche) for 12 h. For nanostring analysis and T cell proliferation assay skin was treated with 1 mg/ml dispase (Invitrogen) to separate epidermis and dermis. Dermal DCs were sorted by fluorescence-activated cell sorting (FACS), epidermal LCs were isolated using CD1a microbeads (Miltenyi Biotec) and a magnet (Stemcell techonologies) with a purity of >90%.

For isolation of mouse skin cells, mice were sacrificed and ears were cut off at the base. Ear skin was split into dorsal and ventral halves and incubated in RPMI+10%FCS containing 1 mg/ml dispase (Invitrogen) for 2 h at 37deg. Epidermis and dermis were separated and digested in 0.2 mg/ml collagenase (Type IV, Sigma) for 2 h at 37deg before passing them through a 70 um filter to obtain a single cell suspension.

Mouse skin-draining auricular lymph nodes were isolated, incubated in medium+0.2 mg/ml collagenase for 30 min and passed through 70 um filter.

BHK-21 and C6/36 cells were purchased from the American Type Culture Collection.

### Virus

For infection experiments the following strains of dengue virus were used: DENV-1 – 08K3126, DENV-2 - TSV-01 or D2Y98P, DENV-3 – VN32/96, and DENV-4 – 2641Y08. All strains are patient isolates that have been passaged in C6/36 mosquito cells for 5–20 passages. D2Y98P used here was plaque-purified after passage 20 and derived from an infectious clone [19]. The enhanced viral RNA synthesis capacity of D2Y98P was mapped to a natural mutation in NS4b. The mutation had no effect on the IFN-inhibiting capacity of the virus [19]. All strains viruses used in the experiments were produced in the C6/36 mosquito cell line. For phagocytosis assays, DENV-3 - VN32/96 was purified with density gradient isolation (Opti-Prep, Sigma) according to manufacturer's protocol. Viral particles were labeled with Alexa-647 fluorescent dye using a protein labeling kit (Molecular Probes) and the excess dye was removed with Amicon protein purification tubes (Millipore) according to manufacturer's protocol. The virus was then inactivated in 2 mM DEPC/PBS-T for 15 min at RT [53].

### Flow cytometry and antibodies

Flow cytometry was performed on an LSRII, FACSCanto, FACS was performed using a FACSAriaII (all Becton Dickinson [BD]). Software analysis was performed with FlowJo (TreeStar).

The following reagents for labeling of human cells were used: Carboxyfluorescein succinimidyl ester (CFSE), fixable live/dead blue dye (Life Science Technologies), anti-CD3 (UCHT1), anti-CD4 (RPA-T4), anti-CD8 (RPA-T8), anti-CD1a (HI149), anti-CD209 (9E9A8), anti-CD206 (15-2) (all from Biolegend), anti-CD11c (B-ly6), AnnexinV Detection Kit, anti-CD45 (HI30), anti-HLA-DR (L243) (all from BD Biosciences), anti-CD141 PE (AD5-14H12) (Miltenyi), anti-CD14 (RMO52) (Beckman Coulter), anti-CCR7 (3D12) (eBioscience), anti-Axl (MAB154) (R&D Systems) and anti-E protein (4G2) (ATCC).

The following antibodies were purchased from Biolegend to label mouse cells: anti-CD45 (30-F11), anti-IAIE (M5/114.15.2), anti-CD11b (M1/70), anti-CD11c (N418), anti-CD326 (EpCAM) (G8.8), anti-CD103 (2E7), anti-Ly6C (HK1.4).

### T cell proliferation assay

Sorted DCs were infected for 2 h and immediately co-cultured with allogeneic CD3^+^ flow-sorted CFSE-labeled T cells from healthy blood donors in a ratio of 1∶10 in 96-well U-bottom plates for 5 days before proliferation was determined by CFSE dilution.

### Chemotaxis assay

Cell migration was assayed in chemotaxis microchamber plates (Neuroprobe) containing a membrane with 5 µm pores. Briefly, medium alone or containing recombinant human CCL19 (20 ng/ml, R&D Systems) was added to the lower chamber. The membrane was placed on top and a cell droplet (containing approximately 250,000 cells) was pipetted on top of the membrane. Plates were incubated for 2 h at 37 degrees C and relative cell numbers of migrated cells were determined using a CellTiter Glo luminescent cell viability assay, read on a GloMax-96 microplate luminometer (both from Promega).

### Nanostring

Nanostring analysis and initial data processing was performed in the nCounter system according to manufacturer's instructions. The human inflammation gene cartridge (GXA-IN1) was used, and based on the data PGK1, TUBB and GAPDH were used as housekeeping controls. Differential expression analysis was determined with a 2-way ANOVA using celltype and infection status as factors in R v2.15.2/Bioconductor. Multiple testing correction was performed using the method of Benjamini and Hochberg. Heat maps were generated using the logarithmically transformed fold changes of averaged normalized counts for each cell population using the non-infected samples as the reference. Visualization of the data and test results were done using TIBCO Spotfire. NCBI accession numbers of all genes are listed in Table S3.

### Quantitation of proteins in culture supernatants

Levels of CCL5 and IFNβ in skin cell supernatants were measured by enzyme-linked immunosorbent assay (ELISA) (both R&D Systems) following the manufacturer's instructions.

### Determination of virus in cell culture supernatant

Virus titer cell culture supernatant was determined by plaque-forming assay using BHK-21 cells as described elsewhere [35]. Briefly, supernatant of infected cells was diluted 10-fold on BHK-21 cells. After 1 h, medium was exchanged for 0.8% methylcellulose in RPMI/10%FCS and plates were incubated for 4 days. Plaque counts were used to calculate viral titer in plaque forming units per ml.

### Viral qPCR

Viral RNA was extracted from cell supernatants using a viral RNA extraction (Roche) according to the manufacturer's protocol and subsequently quantified by real-time qRT-PCR using primers and methods reported previously [54]. Forward primer ACACCACAGAGTTCCATCACAGA, reverse primer CATCTCATTGAAGTCNAGGCC, probe CGATGGARTGCTCTC.

### Binding and DC-SIGN blocking assay

Binding experiments were performed with U937 cells stably expressing DC-SIGN [54]. Virus was incubated with the cells for 1 h at 4°C and cells were subsequently washed with serum-free medium. Non-fluorescently labelled virus was detected with 4G2-A647 anti-E antibody. For DC-SIGN blocking experiments cells were pre-incubated with 20 ug/ml anti-DC-SIGN mAb (clone 120507) or a matched isotype controls (clone 133303) (R&D Systems) for 1 h at 37°C.

## Supporting Information

Figure S1 **Human skin DC gating strategy, intradermal infection and surface molecule expression.** (A) Gating strategy to identify DC subsets after collagenase digestion of healthy human skin tissue: CD14+ (green), LCs (blue), CD1c+ (red) and CD141+ (orange). (B) Macrophage gating strategy, expression of selected markers and infection rate at 24 hpi compared to CD14^+^ dermal DCs. (C) Suspension infection (left) versus intradermal injection (right) of skin DCs infected with DENV-2 (TSV01, MOI5) for 48 h. n = 3 and n = 5, respectively, mean ± SEM.
(TIF)

Figure S2 **Expression of DENV receptors on primary cells and binding of DENV to DC-SIGN.** (A) Binding of DEPC-inactivated fluorescently labeled DENV-3 to DC-SIGN expressed on U937 cells. Cells were pre-incubated with DC-SIGN blocking- or a control Ab or left untreated at 37°C for 1 h and subsequently exposed to the virus at 4°C for 1 h. Mean fluorescence intensity (MFI) was measured by flow cytometry. Two independent experiments were performed in triplicates. mean ± SD (B) Surface expression of DC-SIGN (CD209), MMR (CD206) and Axl on skin DC subsets. One representative of three donors is shown. (C) Binding and blocking of LIVE DENV-2 to DC-SIGN expressed on U937 cells (as described in (A)), one experiment was performed in quadruplicates, mean ± SD.
(TIF)

Figure S3 **Murine skin DC gating strategy and infection of CD45^−^ cells in mouse skin.** (A) Gating strategy to identify DC subsets after collagenase digestion of murine skin tissue in non-treated or DENV-2-infected IFNAR^−/−^ mice at 2 or 4 dpi: Infiltrating monocytes (IAIE^−^Ly6C^+^SSC^lo^, gate 1), CD103^+^ DCs (2), CD11b^−^ DCs (3), EpCAM^+^ LCs (4), CD11b^+^ DCs (5) and monocyte-derived cells (IAIE^+^Ly6C^+^) (6). (B) Presence of DENV E protein was measured in CD45^−^ cells (see (A)) from the epidermis and dermis, 2 and 4 days after infection. One representative results (n = 4–5) is shown.
(TIF)

Table S1 **DENV-infected cells are not apoptotic.** Annexin V stain 48 and 90 hpi, related to Fig. 2F. Mean percentage of two donors per time point from four independent experiments.
(PDF)

Table S2 **CD8+ T cell proliferation is not altered by infection of DC subsets.** CD8+ T cell proliferation (related to Fig. 4A and B). Mean of 3–4 donors ± SEM.
(PDF)

Table S3 **List of genes and corresponding accession numbers from nanostring analysis in** **Fig. 5A** **.**
(PDF)
