# Supplementary figures and images for: Selective Susceptibility of Human Skin Antigen Presenting Cells to Productive Dengue Virus Infection
Source: PLoS Pathog. 2014 Dec 4;10(12):e1004548. doi: 10.1371/journal.ppat.1004548 (PMC4256468; doi:10.1371/journal.ppat.1004548)

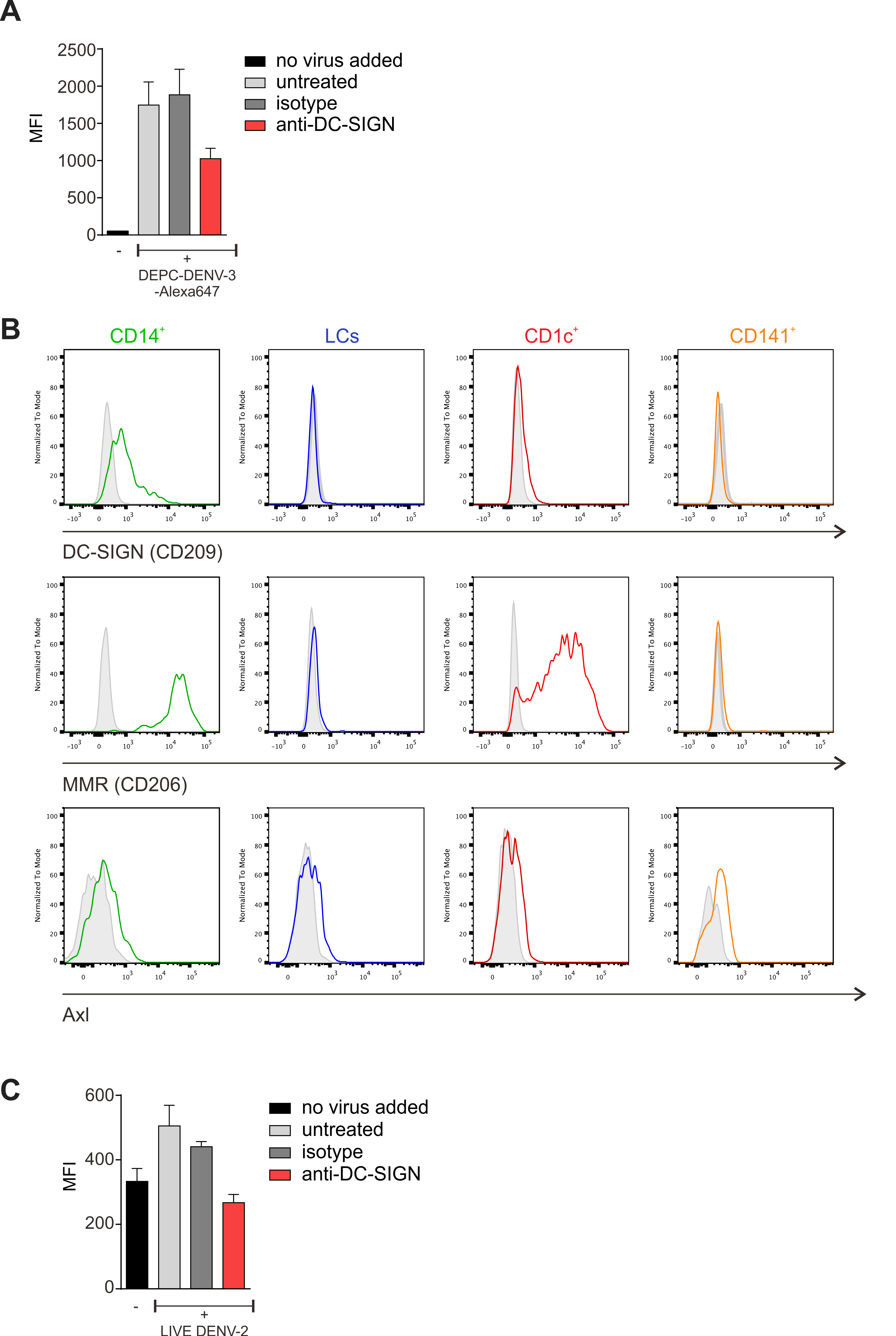

Supplement: Figure S2 — Expression of DENV receptors on primary cells and binding of DENV to DC-SIGN. (A) Binding of DEPC-inactivated fluorescently labeled DENV-3 to DC-SIGN expressed on U937 cells. Cells were pre-incubated with DC-SIGN blocking- or a control Ab or left untreated at 37°C for 1 h and subsequently exposed to the virus at 4°C for 1 h. Mean fluorescence intensity (MFI) was measured by flow cytometry. Two independent experiments were performed in triplicates. mean ± SD (B) Surface expression of DC-SIGN (CD209), MMR (CD206) and Axl on skin DC subsets. One representative of three donors is shown. (C) Binding and blocking of LIVE DENV-2 to DC-SIGN expressed on U937 cells (as described in (A)), one experiment was performed in quadruplicates, mean ± SD. (TIF) [file ppat.1004548.s002.tif]
