# Supplementary material for: Selective Susceptibility of Human Skin Antigen Presenting Cells to Productive Dengue Virus Infection
Source: PLoS Pathog. 2014 Dec 4;10(12):e1004548. doi: 10.1371/journal.ppat.1004548 (PMC4256468; doi:10.1371/journal.ppat.1004548)
Supplement: Table S1 — DENV-infected cells are not apoptotic. Annexin V stain 48 and 90 hpi, related to Fig. 2F. Mean percentage of two donors per time point from four independent experiments. (PDF) [file ppat.1004548.s004.pdf]

		CD14+				LCs				CD1c+			
		Annexin + 4G2 -	Annexin + 4G2 +	Annexin - 4G2 +	Annexin - 4G2 -	Annexin + 4G2 -	Annexin + 4G2 +	Annexin - 4G2 +	Annexin - 4G2 -	Annexin + 4G2 -	Annexin + 4G2 +	Annexin - 4G2 +	Annexin - 4G2 -
**48hpi**	mock	33.9	1.4	8.1	56.7	59.0	0.5	2.5	38.0	9.2	0.2	2.3	88.3
	DENV-2	35.2	8.5	22.6	33.8	57.0	1.6	16.3	25.2	9.2	0.4	15.3	75.0
**90hpi**	mock	15.1	0.4	0.1	84.5	44.2	0.3	0.0	55.5	8.1	0.0	0.0	91.9
	DENV-2	11.9	5.7	45.7	36.7	34.3	2.3	12.0	51.4	7.3	0.5	18.3	73.9

Table S1. DENV-infected cells are not apoptotic

Annexin V stain 48 and 90hpi, related to Fig.2F. Mean percentage of two donors per time point from four independent experiments.
