# Supplementary material for: Selective Susceptibility of Human Skin Antigen Presenting Cells to Productive Dengue Virus Infection
Source: PLoS Pathog. 2014 Dec 4;10(12):e1004548. doi: 10.1371/journal.ppat.1004548 (PMC4256468; doi:10.1371/journal.ppat.1004548)
Supplement: Table S2 — CD8+ T cell proliferation is not altered by infection of DC subsets. CD8+ T cell proliferation (related to Fig. 4A and B). Mean of 3–4 donors ± SEM. (PDF) [file ppat.1004548.s005.pdf]

	$CD14^+$	LCs	$CD1c^+$
**mock**	2.3±0.15	1.95±0.3	3.76±1.5
**DENV-2**	2.1±0.4	2.4±1.0	3.5±1.6

Table S2. $CD8^+$ T cell proliferation is not altered by infection of DC subsets

$CD8^+$ T cell proliferation (related to Fig.4A and B). Mean of 3-4 donors ± SEM.

	CD14+	LCs	CD1c+
**mock**	2.3±0.15	1.95±0.3	3.76±1.5
**DENV-2**	2.1±0.4	2.4±1.0	3.5±1.6

Supporting Table S2. CD8+ T cell proliferation is not altered by infection of DC subsets

CD8+ T cell proliferation (related to Fig.4A and B). Mean of 3-4 donors ± SEM.
