# Supplementary material for: Selective Susceptibility of Human Skin Antigen Presenting Cells to Productive Dengue Virus Infection
Source: PLoS Pathog. 2014 Dec 4;10(12):e1004548. doi: 10.1371/journal.ppat.1004548 (PMC4256468; doi:10.1371/journal.ppat.1004548)
Supplement: Table S3 — List of genes and corresponding accession numbers from nanostring analysis in Fig. 5A . (PDF) [file ppat.1004548.s006.pdf]

Gene	NCBI Accession number
IFNB1	NM_002176.2
CCL5	NM_002985.2
STAT1	NM_007315.2
LY96	NM_015364.2
CCL3	NM_002983.2
IL23A	NM_016584.2
CCL19	NM_006274.2
RIPK2	NM_003821.5
TNF	NM_000594.2
IFNA1	NM_024013.1
IL3	NM_000588.3
MAPK3	NM_001040056.1
FASLG	NM_000639.1
CRP	NM_000567.2
CXCL2	NM_002089.1
TLR3	NM_003265.2
CXCL10	NM_001565.1
DDIT3	NM_004083.4
TLR1	NM_003263.3
RIPK1	NM_003804.3

Table S3. List of genes and corresponding accession numbers from nanostring analysis in Fig.5A
